# Silicon-Vacancy Nanodiamonds as High Performance Near-Infrared Emitters for Live-Cell Dual-Color Imaging and Thermometry

**DOI:** 10.1021/acs.nanolett.2c00040

**Published:** 2022-03-15

**Authors:** Weina Liu, Md Noor A. Alam, Yan Liu, Viatcheslav N. Agafonov, Haoyuan Qi, Kaloian Koynov, Valery A. Davydov, Rustem Uzbekov, Ute Kaiser, Theo Lasser, Fedor Jelezko, Anna Ermakova, Tanja Weil

**Affiliations:** † Max-Planck-Institute for Polymer Research, Ackermannweg 10, 55128 Mainz, Germany; ‡ Institute of Inorganic Chemistry I, Ulm University, Albert-Einstein-Allee 11, 89081 Ulm, Germany; § Institute of Materials, École Polytechnique Fédérale de Lausanne, Station 12, 1015 Lausanne, Switzerland; ∥ Beijing Academy of Quantum Information Sciences, No.10 Xi-bei-wang East Road, 100193 Beijing, China; ⊥ Institute for Quantum Optics, Ulm University, Albert-Einstein-Allee 11, 89081 Ulm, Germany; # GREMAN, UMR CNRS-7347, Université de Tours, 37200 Tours, France; ∇ Central Facility for Electron Microscopy, Ulm University, Albert-Einstein-Allee 11, 89081 Ulm, Germany; ¶ Center for Advancing Electronics Dresden (cfaed) and Food Chemistry, Technical University of Dresden, 01069 Dresden, Germany; □ L. F. Vereshchagin Institute for High Pressure Physics, The Russian Academy of Sciences, Troitsk, Moscow 108840, Russia; ● Laboratoire Biologie Cellulaire et Microscopie Electronique, Faculté de Médecine, Université François Rabelais, 37032 Tours, France; + Faculty of Bioengineering and Bioinformatics, Moscow State University, Leninskye gory 73, Moscow 119992, Russia; ⬡ Institute for Physics, Johannes Gutenberg University Mainz, Staudingerweg 7, 55128 Mainz, Germany

**Keywords:** Nanodiamond, silicon vacancy color center, near-infrared cellular imaging, live cell particle tracking, thermometry

## Abstract

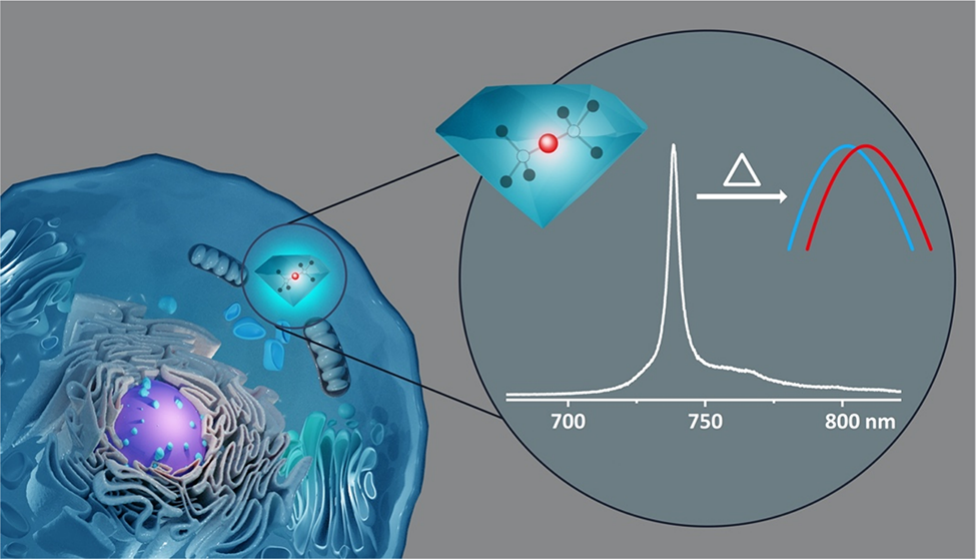

Nanodiamonds (NDs) with color centers are excellent emitters for various bioimaging and quantum biosensing applications. In our work, we explore new applications of NDs with silicon-vacancy centers (SiV) obtained by high-pressure high-temperature (HPHT) synthesis based on metal-catalyst-free growth. They are coated with a polypeptide biopolymer, which is essential for efficient cellular uptake. The unique optical properties of NDs with SiV are their high photostability and narrow emission in the near-infrared region. Our results demonstrate for the first time that NDs with SiV allow live-cell dual-color imaging and intracellular tracking. Also, intracellular thermometry and challenges associated with SiV atomic defects in NDs are investigated and discussed for the first time. NDs with SiV nanoemitters provide new avenues for live-cell bioimaging, diagnostic (SiV as a nanosized thermometer), and theranostic (nanodiamonds as drug carrier) applications.

Currently, fluorescent molecules are mostly used as labels for intracellular imaging. However, their applications for time-laps monitoring are limited by a fast-photobleaching time. A promising alternative are nanodiamonds (NDs) with color centers that demonstrate high photostability.^1^ Depending on the type of color center, they can be used for bioimaging and sensing applications, such as super-resolution imaging or nanoscale magnetometry and thermometry.^2−4^ The most investigated diamond color center is the nitrogen-vacancy center (NV), which consists of a substitutional nitrogen atom next to a carbon vacancy. NDs with NV (ND-NV) are commercially available and can be produced in different sizes and with varying numbers of NV.^5^ The NV reveals two charge states: the neutral NV^0^ or the negative NV^–^ that both have stable fluorescence, but only the NV^–^ is suitable for sensing application.^6^ Zero phonon lines (ZPLs) of NV^0^ and NV^–^ are accompanied by broad phonon sidebands, leading to broad emission spanning from ∼575 nm (NV^0^) or 637 nm (NV^–^) to 800 nm.^6^ Although ND-NV can be used for long-term bioimaging studies, their spectrum partly overlaps with many optical markers and cellular autofluorescence so that dual/multicolor imaging remains challenging. Conversely, NDs with negatively charged silicon-vacancy centers (SiV) have recently received attention as high-performance bioimaging probes due to their attractive optical properties with sharp near-infrared (NIR) emission.^7^ The silicon atom with its larger size compared to the carbon atom size replaces two carbon atoms and is located between these two vacancies (Figure 1a). This divacancy structure of SiV has inversion symmetry resulting in low sensitivity to strain and contributes to a narrowing of the fluorescence.^7^ Due to the low electron–phonon coupling, more than 70% of the SiV emission is dominated by the sharp ZPL at ∼738 nm with the full width at half-maximum (fwhm) of approximately 4 nm.^7^ The NIR emission of ND-SiV allows deeper tissue penetration and in vivo imaging.^8,9^ Moreover, the ZPL peak position of SiV has a temperature signature, which is linearly correlated to temperature changes in the range of 295 ± 5 K with subkelvin sensitivity.^10^ The combination of NIR emission, narrow bandwidth, high photo- and chemical stability, and a temperature-dependent ZPL^11,12^ renders ND-SiV as promising candidates for bioimaging and thermometry in life sciences.

**Figure 1:**
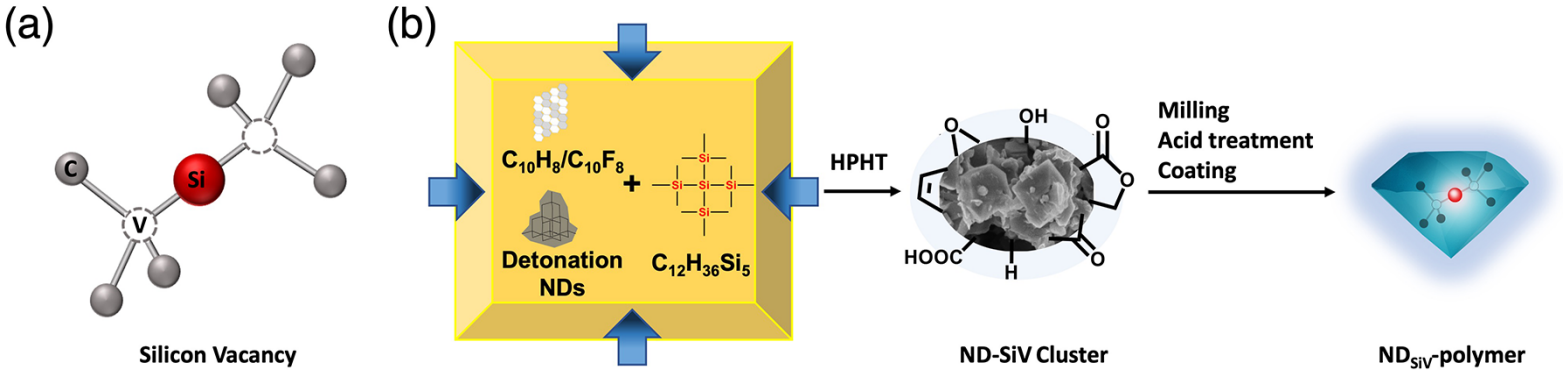
(a) Atomic structure of the SiV center displayed by one silicon atom (Si) with two adjacent atom vacancies (V) in the diamond lattice of carbon atoms (C). (b) Schematic presentation of ND-SiV HPHT synthesis and modification by coating.

In this work, we report the production and functionalization of ND-SiV for live-cell dual-color imaging, thermometry, and tracking. We optimize the metal-catalyst-free high-pressure high-temperature (HPHT) approach to synthesize ND-SiV with radii of about 50 nm and without the presence of NV. The ND-SiV surface is coated by a protein-derived biopolymer on the basis of multiple electrostatic interactions resulting in nanoparticles with enhanced colloidal stability. These coated ND-SiV reveal a good uptake by HeLa and A549 cells based on an endocytosis mechanism. For the first time, HPHT ND-SiV have been used for live-cell dual-color imaging on the basis of their sharp NIR emission and high photostability. Moreover, the first intracellular thermometry by ND-SiV with radii 50 nm and less has been demonstrated.

Traditionally, NDs are synthesized by HPHT growth in the presence of transition metal catalysts or chemical vapor deposition (CVD) growth.^13^ The color centers can be introduced by adding impurities during diamond growth or by ion implantation. The metal-catalyst-free synthesis is preferable for fluorescent NDs production since metal atoms can introduce additional defects into the crystal structure, which can deteriorate the properties of the color centers. Such a method is based on the conversion of organic and heteroorganic solids into diamond.^14,15^ This technique allows controlling ND sizes and color centers concentrations.^16,17^ Herein, we present the production of ND-SiV from a homogeneous mixture of naphthalene (C_10_H_8_), octafluoronaphthalene (C_10_F_8_), detonation NDs (3–4 nm), and tetrakis(trimethylsilyl)silane (C_12_H_36_Si_5_), which is used as the doping component (Figure 1b). The introduction of fluorine-containing compounds into the growth leads to the reduction of NV in NDs.^15,16,18^ Detonation NDs are introduced as seeds in the HPHT reaction to obtain higher yields of nanosized fraction of diamond. The homogeneous mixture of the initial components of the growth system is cold-pressed as a tablet (5 mm diameter and 4 mm height) and placed into a graphite container, which simultaneously serves as a heater for the high-pressure Toroid-type apparatus. The HPHT growth comprises the following steps: (1) reaching high pressure (8.0 GPa) at room temperature, (2) heating to high temperature (∼1400 °C) for diamond formation, and (3) an isothermal exposure for short time (3 s). Then the temperature is decreased to room temperature, while the pressure remains high. The applied conditions trigger ND formation inside the initial tablet of the pressed components. Five batches are synthesized under the same conditions and combined to maximize the amount of ND powder. The tablets are milled by steel balls into micro- and nanoparticles before chemical cleaning. A primary cleaning step with HNO_3_:HClO_4_:H_2_SO_4_ at 230 °C for 5 h generates a powder, which is then neutralized with NH_4_OH buffer, washed, and dried as depicted in Scheme S1.

Scanning and transmission electron microscopy (SEM and TEM, Figures S1 and S2) show the formation of nano- and microdiamonds with cuboctahedral shapes. The photoluminescence (PL) measurements reveal a sharp SiV spectrum (Figure 2e), without the presence of NV due to the application of C_10_F_8_ as a starting material during synthesis. Alternatively, NDs synthesized without C_10_F_8_ show strong NV and SiV emissions in their PL spectra (Figure S3). The demonstrated HPHT ND-SiV synthesis offers several distinct advantages: (1) no metal catalyst is employed that can remain as impurities in the NDs, (2) no postprocessing by irradiation or annealing is required to activate the color centers, and (3) the method is scalable up to several milligram quantities, which would allow extensive cell studies with high reproducibility in the same batch.

**Figure 2:**
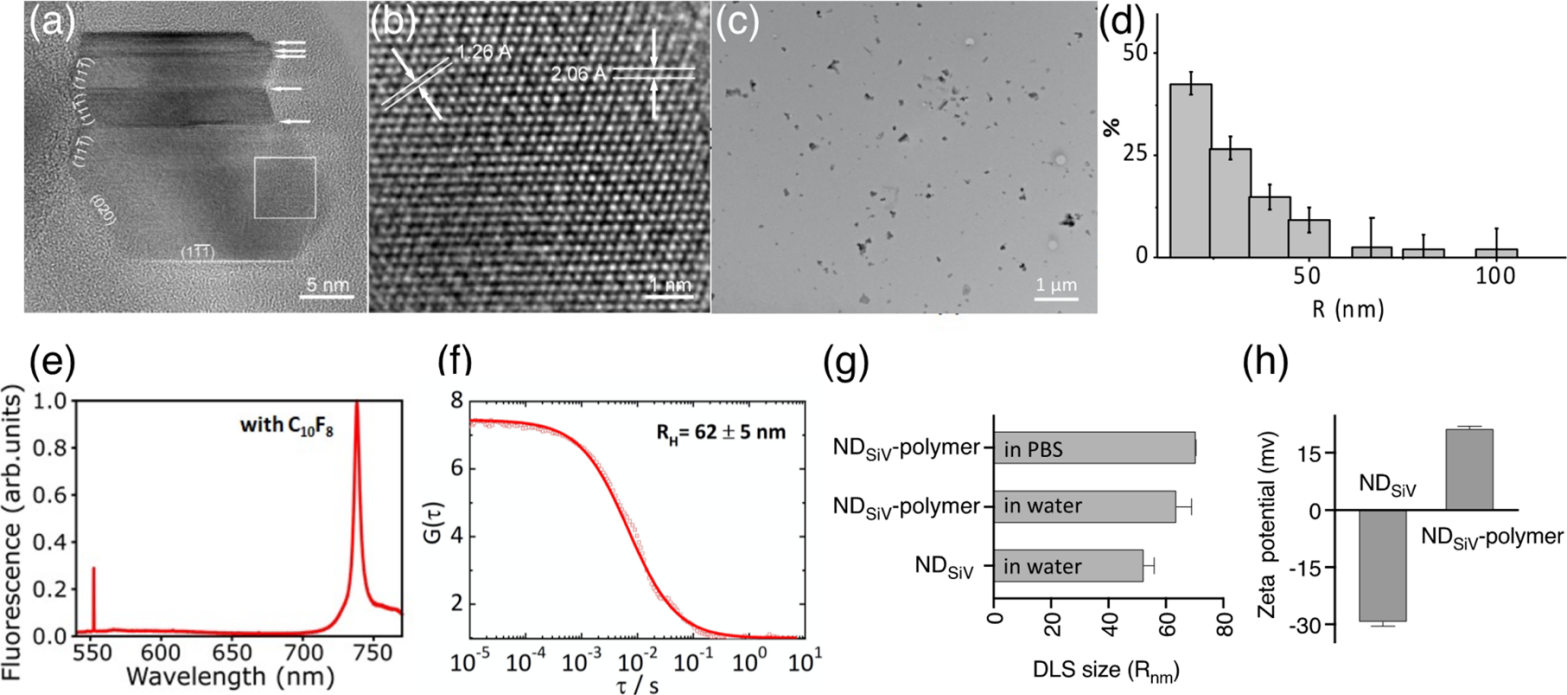
Characterization of ND-SiV and ND_SiV_-polymer. (a) [01̅1] AC-HRTEM image of ND-SiV consisting of crystalline domains separated by twin boundaries (marked by arrows). (b) Magnified image from the boxed region in (a), showing the distances *d* between the diamond lattice planes: (111) (*d* = 2.06 Å) and (022) (*d* = 1.26 Å). (c) TEM image of the coated ND_SiV_-polymer. Small clusters can be observed from the TEM images, but most of the NDs are discrete nanoparticles. (d) Histogram of NDs radius, quantification of 108 NDs from (c). (e) PL spectra of HPHT ND-SiV synthesized with C_10_F_8_. (f) FCS autocorrelation curves of ND-SiV in water solution with the obtained hydrodynamic radii. (g) DLS radius of ND-SiV in water and ND_SiV_-polymer in water and PBS buffer. (h) ζ potential of ND-SiV and ND_SiV_-polymer.

Surface cleaning and oxidization are accomplished by combining acid treatment (HNO_3_:H_2_SO_4_:HClO_4_, ratio 1:1:1, at 90 °C for 8 h) and sonication (Scheme S1). We obtain about 5 mg of a stable ND-SiV suspension in water without clusters with polar carboxylic acid surface groups,^19^ allowing further chemical modifications.^20^ The dimensions of ND-SiV in water are determined by fluorescence correlation spectroscopy (FCS)^21^ and dynamic light scattering measurement (DLS). Nanoparticles with average hydrodynamic radii of 62 ± 5 nm (FCS, Figure 2f) and 52.3 ± 3.6 nm (DLS, Figure 2g) with a polydispersity index (PDI) of 0.16 (Figure S7) are recorded as single nanoparticles (according to FCS) and no ND clusters are observed. Noteworthy, the FCS method detects only fluorescent nanoparticles, whereas DLS determines all NDs in the solution, which could be a reason for the small differences in ND sizes measured by these two methods. Besides, there is a difference in PDI of FCS and DLS measurements.^22^ After the acid treatment, ND-SiV exhibit a negative ζ-potential with a single peak distribution (ζ = −29.33 mV, Figure 2h and Figures S8–S10).

The application of ND-SiV for cellular studies requires surface coating that imparts colloidal stability in cell media and allows cellular uptake and trafficking to cellular compartments with low cellular toxicity. We have previously reported the conversion of plasma proteins into biocompatible ND surface coatings that have been applied in vitro and in vivo.^23^ Herein, the human serum albumin (HSA) has been chemically modified by reacting ethylenediamine groups with the carboxylic acid surface groups of aspartic acid and glutamic acid residues, yielding cationic HSA (cHSA, cationazation) as described previously and as depicted in Figure S4.^23−25^ cHSA with multiple additional amino groups provides multiple positive net charges, which are required for the subsequent formation of stable complexes with a negatively charged surface of ND-SiV by electrostatic interactions. Hydrophilic polyethylene glycol (PEG) chains (average molecular weight of 2000 Da) are conjugated to cHSA to improve the colloidal stability of coated ND-SiV (cHSA-PEO, PEGlytion). Next, the polypeptide backbone of cHSA-PEO is unfolded by the reduction of disulfide bridges. The generated free sulfhydryl groups are capped with *N*-(2-aminoethyl)maleimide to obtain the stable single-chain positively charged biopolymer (dcHSA-PEO, denaturation). The synthesis and characterization of the biopolymer dcHSA-PEO have been reported previously^23^ and are included in the SI (Figure S11 and Figure S12).

ND-SiV are coated with dcHSA-PEO by first diluting the negatively charged nanoparticles in boric acid buffer (0.05 mg mL^–1^, 20 mL, pH = 8.4) and then titrating ND solution into dcHSA-PEO solution (0.2 mg mL^–1^, dispersed in the same boric acid buffer, 20 mL). The mixture is stirred overnight and coating proceeds by electrostatic adsorption of the positively charged modified proteins to the ND surface. After ultrafiltration (cutoff 30 KD) and centrifugation (17 000 rpm, 30 min, 3 times), the ND mixture is concentrated, and unbound dcHSA-PEO biopolymer is removed. About 1 mg (50% yield) of coated ND-SiV is obtained, termed ND_SiV_-polymer (Figure 1b). Figure 2a shows the aberration-corrected high-resolution TEM (AC-HRTEM) image of ND_SiV_-polymer in the [01̅1] projection. Highly crystalline ND_SiV_-polymer is observed exhibiting sharp edges along the main crystallographic orientations. In the magnified image (Figure 2b), the (111) and (022) lattice planes of the diamond are clearly resolved. Residual amounts of amorphous and nondiamond nanoparticles are also observed via AC-HRTEM (Figure S5), which could not be removed by the acid processing. However, the X-ray diffraction patterns (XRD) of the ND-SiV raw material indicate the typical diamond spectrum with reflections at (111) and (220) (Figure S6).

A uniform and discrete distribution of ND_SiV_-polymer is determined by TEM (Figure 2c), with an average radius of 31.6 nm (histogram in Figure 2d) after analyzing about 108 NDs. After the protein–polymer encapsulation, DLS reveals a hydrodynamic radius of ND_SiV_-polymer in the water of about 63.6 ± 5.3 nm (PDI = 0.1) corresponding to an increase of about 11 nm due to the protein–polymer shell (Figure 2g). ND_SiV_-polymer appears colloidally stable also in phosphate-buffered saline (PBS, pH = 7.4), and the radius increases only slightly to 70 nm (PDI = 0.08, DSL measurements). The surface charges of ND_SiV_-polymer are positive (ζ = 21.3 mV) due to the polycationic biopolymer coating dcHSA-PEO. Nanoparticle surface coatings with positive net charges often facilitate cellular uptake due to electrostatic interactions with the negatively charged cellular membranes (Figure 2g,h).^18,23^

ND_SiV_-polymer is applied for live-cell imaging in HeLa cells used as a model cell line. ND_SiV_-polymer (0.02 mg mL^–1^) is incubated for 24 h to enable cellular uptake, which is recorded by a commercial confocal microscope. In a previous study, ND-SiV powder prepared by the CVD method or by Si implantation has been directly added to cells^26^ without stabilizing surface modifications. In these cases, even after several days of incubation, either limited internalization (NDs prepared by CVD method) or only NDs (prepared by Si implantation) aggregation at the cell surface is observed. In the present study, ND_SiV_-polymer is taken up and appears homogeneously distributed within cells (Figure 3a and Figure S13). Due to the high index of refraction, these ND_SiV_-polymers act as strong light scatterers, allowing us to distinguish NDs from the background fluorescence of HeLa cells, which proves to be very helpful for further multiple stained bioimaging. According to Figure 3a, the images taken in the reflection mode of ND_SiV_-polymer show a good localization match with ND_SiV_-polymer fluorescence images (colocalization coefficient 0.6), indicating that most NDs contain SiV. The nonoverlapping portion is attributed to a small fraction of NDs lacking SiV since the reflection imaging depicts all NDs, and fluorescence imaging shows only NDs with color centers. It has been reported that SiV within NDs could blink and bleach by pathways still not fully understood.^27^ Furthermore, a time series scan has been processed to evaluate the photostability of ND_SiV_-polymer. Some of the fluorescent points bleach after three scanning sweeps (Figure S14a), but the remaining emitters show high stability (Figure S14b), making them suitable for cellular imaging and tracking.

**Figure 3:**
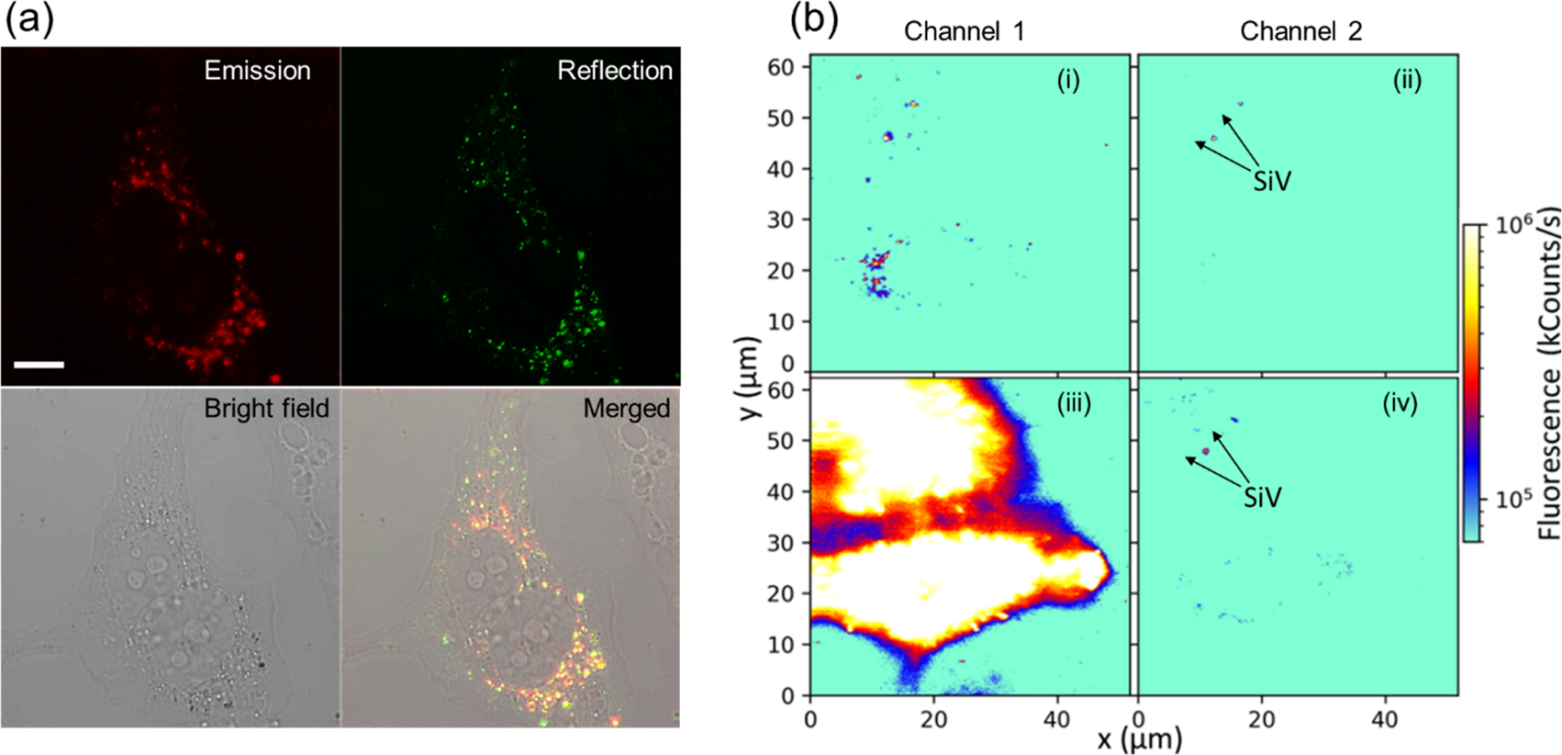
ND_SiV_-polymer for dual-color cell imaging. (a) Confocal microscopy cell images showing efficient cell uptake. Emission and reflection channels demonstrated very good colocolization (λ_ex_ = 561 nm, λ_em_ = 700–758 nm, λ_re_ = 556–566 nm, scale bar = 10 μm). (b) Fluorescence cell images obtained by a customized confocal microscope (λ_ex_ = 532 nm) with two detection channels (1 – λ_em_ = 575 nm and longer, 2 – λ_em_ = 720–760 nm).

HeLa cells incubated with ND_SiV_-polymer have been investigated by a customized confocal microscope (Figure 3b). The cell culture medium is replaced with a phenol red-free buffer to avoid additional fluorescence. Laser excitation at 532 nm with the power of 200 μW (measured before the objective) is performed, and the fluorescence is recorded simultaneously by two different detection channels. Channel 1 with a long-pass filter detects the light with a wavelength longer than 575 nm (Figure 3b (i, iii)). Channel 2 has a band-pass filter to register emitted light in the range 720–760 nm corresponding to the SiV ZPL. The initial images of HeLa cells incubated with ND_SiV_-polymer are presented in Figure 3b (i, ii). In channel 1 (Figure 3b (i)), we observe SiV together with cellular autofluorescence. To prove the presence and position of ND_SiV_-polymer only, channel 2 is successfully used (Figure 3b (ii)) where cellular autofluorescence is filtered. Cellular autofluorescence is very weak; therefore, the CellMask green dye is added for better cell visualization (Figure 3b (iii, iv)). The signal from CellMask green is a few orders of magnitude higher than that from SiV due to the high number of dye molecules in the focal spot (Figure 3b (iii)). However, the presence of membrane dyes does not interfere with the imaging of ND_SiV_-polymer in channel 2 (Figure 3b (iv)). These experiments prove the suitability of ND-SiV for dual-color live-cell imaging because the sharp NIR ZPL emission of ND-SiV could be easily separated from many dyes and drugs.

In the next step, we have studied thermometry capabilities of ND_SiV_-polymer. In bulk diamond with SiV, a ZPL shift of about 0.0124 nm per 1 K is demonstrated.^10^ In the same work, a change in the intensity of SiV fluorescence in NDs with a size of 200 nm is shown. NDs with NV are used for intracellular thermometry,^28^ but such experiments require microwave field while SiV offers pure optical measurements. However, NDs with SiV have not been tested yet for thermometry by ZPL shift measurements. Herein, we analyze spectra of 12 ND-SiV spots in water at temperatures ranging from 25 to 37.5 °C with a step of 2.5 °C. The experiments are performed with a customized confocal microscope upgraded with a cell incubator (OkoLab, H301-MINI) with temperature stabilization for 25–40 °C with an accuracy of 0.1 °C. At 25 °C (Figure 4a) the ND-SiV spectra reveal varying ZPL peak positions and widths (Figure 4b,c, Figure S15a,b), which could be a result of the crystal strain due to additional diamond defects, varying NDs shapes and morphologies, arbitrary positions of SiV and their number per nanocrystal, and the number of NDs in one spot.^29^ For a significant temperature increase from 25 to 37.5 °C, we observe the red shift of the SiV ZPL for each spot (Figure 4b, Figure S15a). However, for some spots at intermediate temperatures between 25 and 37.5 °C, a deviation of the ZPL shift is observed (Figure S15a). Particularly, only five out of 12 ND-SiV spots that had a ZPL peak position below 738.20 nm and a fwhm smaller than 4.6 nm show a linear red shift of the ZPL at Δλ/Δ*T* = ∼0.011–0.013 nm/K (deviation ≤8.42%) for each measured temperature (Figure 4b). The remaining seven spots with ZPL peaks above 738.20 nm and fwhm larger than 4.6 nm reveal a strong deviation of the ZPL shift for small temperature steps (Figure S15a). Such behavior relates to crystal strain, which varies from NDs to NDs due to different defects, SiV location, and ND shapes. On the basis of the obtained results, we can conclude that thermometry with ND-SiV is possible. Nevertheless, the precise detection of small temperature variations might be challenging to achieve, and the initial properties of ND-SiV should be evaluated prior to thermometry measurements.

**Figure 4:**
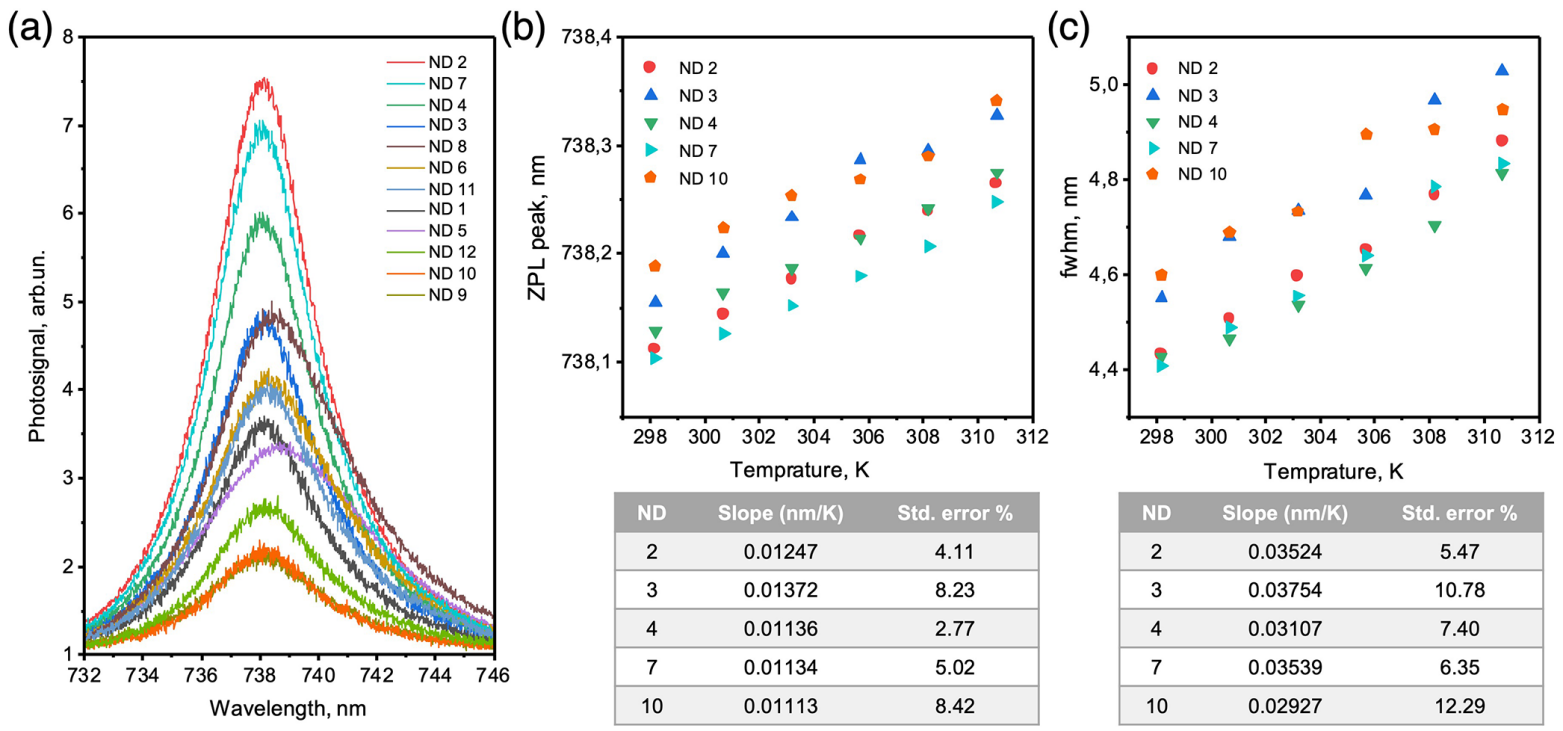
Thermal resonance of ND-SiV. (a) ZPL of 12 ND-SiV nanoparticles at 25 °C. (b) Positions of ZPL peaks of five ND_SiV_ nanoparticles with a linear shift in the temperature range from 25 to 37.5 °C with low deviation. (c) FWHM of ZPL spectra for the five ND_SiV_ nanoparticles with a linear broadening in the temperature range from 25 to 37.5 °C with low deviation.

ND_SiV_-polymer nanoparticles have been tested for intracellular thermometry in fixed and living A549 cells, using fixed cells as a control to reduce the free motion of NDs compared with living cells (Figure 5a). We have investigated the effect of the cellular environment on ND-SiV fluorescence since even low cellular autofluorescence can affect the detected SiV spectra and have an impact on thermometry. Within cells, the observed ND_SiV_-polymer signals usually originate from ND clusters inside intracellular vesicles.^30^ Unfortunately, all tested ND_SiV_-polymer spots within cells have ZPL peaks above 738.2 nm at 25 °C, which are not suitable for precise thermometry in the cells. Nevertheless, all the measured spots reveal red shifts of ZPL peak positions (Figure S17). One of the measured spots of ND_SiV_-polymer in living cells demonstrates a red shift of about ∼0.06218 nm (average for seven measurements) (Figure 5b, Figure S17) after the temperature is increased from 25 to 37 °C. The live-cell thermometry with ND-SiV requires not only sensing but also a possibility to track the NDs. As a preliminary attempt, we have tracked 135 single ND_SiV_-polymer spots within living HeLa cells for 90 min each with refocusing intervals of 40 s. The representative trajectory of one ND_SiV_ spot is shown in Figure 5c. The tracking measurements are accomplished by the fluorescence intensities of ND_SiV_-polymer during the tracking experiments. The fluorescence intensities of tracked ND_SiV_-polymer remain relatively stable (Figure 5c) with low fluctuations. These fluctuations could be attributed to the fast diffusion from the focal point of the objective or to rotational movement induced by different excitation efficiencies during tracking. The additional tracked NDs with representative trajectories and intensities are depicted in Figure S18a,b. The presence of SiV is proven by spectral measurements (Figure S18c). Any significant decrease in the fluorescence intensities is not observed that allows ND_SiV_-polymer for long-term cellular tracking.

**Figure 5:**
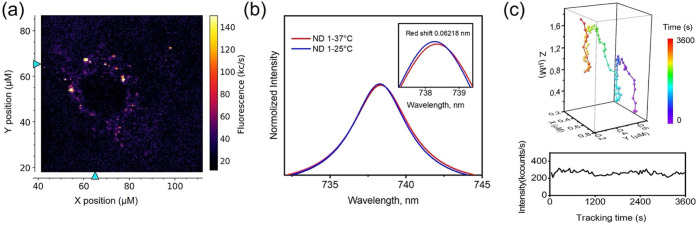
ND_SiV_-polymer for living cell thermometry and intracellular tracking. (a) Custom-built confocal image of a living A549 cell with uptaken ND_SiV_-polymer nanoparticles. (b) Position of ZPL peaks of ND_SiV_-polymer at 25 and 37 °C. (c) Trajectory of ND_SiV_-polymer tracked in intracellular space.

We have reported live-cell dual-color imaging, thermometry, and tracking applications of NDs containing only SiV and no other color centers produced by the improved metal-catalyst-free HPHT method. In this way, NIR emitters are obtained with a single sharp emission signal. These ND-SiV are coated with a protein-derived biopolymer that imparts colloidal stability in water, buffer, and cell media. NIR fluorescence, a sharp ZPL, and high fluorescence stability are key characteristics of these nanoemitters, qualifying them for living cell imaging and tracking. For the first time, HPHT ND-SiV are observed in dual-color imaging and tracking experiments for up to 90 min inside living cells without photobleaching. Thermometry is investigated for the first time with small ND-SiV in water and cells, and a new singularity of ZPL shift with heat is found. We envision that ND-SiV represent a powerful tool for intracellular imaging,^31^ all-optical thermometry,^10^ and tracking,^32^ which renders them attractive for biological studies. However, thermometry with ND-SiV still requires deep and multidimensional investigations. Since NDs can be also used for drug delivery,^23^ a combination of all demonstrated properties of the ND-SiV system paves the way toward theranostic applications.

## Supporting Information Available

Experimental methods of ND_SiV_ acid treatment, of polymer preparation, and of polymer coating of ND_SiV_; characterization of ND_SiV_-polymer; live cell imaging by a confocal microscope; dual-color cell imaging by a customized confocal microscope; thermometry in fixed and living cells with ND_SiV_-polymer; ND tracking with a customized confocal microscope; figures of SEM and TEM images, PL spectra, three-step synthesis, XRD patterns, DLS size, ζ potential, MALDI-TOF spectra, ZPL spectra, scheme of the customized confocal microscope, and thermosensing and cellular tracking measurements (PDF)
