# Supplementary material for: Silicon-Vacancy Nanodiamonds as High Performance Near-Infrared Emitters for Live-Cell Dual-Color Imaging and Thermometry
Source: Nano Lett. 2022 Mar 15;22(7):2881–8. doi: 10.1021/acs.nanolett.2c00040 (PMC9011402; doi:10.1021/acs.nanolett.2c00040)
Supplement: Supplementary file 1 — nl2c00040_si_001.pdf [file nl2c00040_si_001.pdf]

Silicon-Vacancy Nanodiamonds as High Performance Near-Infrared Emitters for Live-Cell Dual-Color Imaging and Thermometry

Weina Liu†∇┤°, Md Noor A Alam†∇°, Yan Liu☐§, Viatcheslav N. Agafonov⊥, Haoyuan Qi‖‡, Kaloian Koynov†, Valery A. Davydov#, Rustem Uzbekov○+, Ute Kaiser‖, Theo Lasser†, Fedor Jelezko§, Anna Ermakova†⫰* and Tanja Weil†∇**

†Max-Planck-Institute for Polymer Research, Ackermannweg 10, 55128 Mainz, Germany

∇Institute of Inorganic Chemistry I, Ulm University, Albert-Einstein-Allee 11, 89081 Ulm, Germany

┤Institute of Materials, École Polytechnique Fédérale de Lausanne, Station 12, 1015 Lausanne, Switzerland

☐ Beijing Academy of Quantum Information Sciences, No.10 Xi-bei-wang East Road, 100193 Beijing, China

§Institute for Quantum Optics, Ulm University, Albert-Einstein-Allee 11, 89081 Ulm, Germany

⊥GREMAN, UMR CNRS-7347, Université de Tours, 37200, Tours, France

‖Central Facility for Electron Microscopy, Ulm University, Albert-Einstein-Allee 11, 89081 Ulm, Germany

‡Center for Advancing Electronics Dresden (cfaed) and Food Chemistry, Technical University of Dresden, 01069 Dresden, Germany

#L.F. Vereshchagin Institute for High Pressure Physics, The Russian Academy of Sciences, Troitsk, Moscow, 108840, Russia

○Laboratoire Biologie Cellulaire et Microscopie Electronique, Faculté de Médecine, Université François Rabelais, 37032 Tours, France

+Faculty of Bioengineering and Bioinformatics, Moscow State University, 119992, Leninskye gory 73, Moscow, Russia

⸸ Institute for Physics, Johannes Gutenberg University Mainz, Staudingerweg 7, 55128 Mainz, Germany

° Contributed equally

* Corresponding authors

Experiments

Acid cleaning, sonication, and ultrasonication treatment

Nanodiamond (25 mg) was mixed with concentrated acid mixture HNO_3 : H_2SO_4 : $HClO_4$ (6 mL, volume ratio 1:1:1) in a 50 mL flask with a connected condenser. Water bath sonication was applied for 15 min to dissociate NDs from aggregates. Subsequently, the flask with nanodiamond and acid mixture was heated in an oil bath to 90 °C and refluxed for 35 min for the surface oxidation of nanodiamond. Following, the oil bath was removed to cool down the

flask for 20 min to the room temperature. The three steps of water bath sonication-oil bath heating-cooling were processed for eight circles. Afterward 10 mL Milli-Q water was drop added to NDs and acid mixture under slowly stirring to dilute the acid. Another 20 mL water was added to the NDs to further dilute the acid. Then the mixture was transferred to a 50 mL centrifugation tube and centrifuged to separated NDs from the acid mixture (885 RPM, 30 min, 4 °C). Further, the acid mixture was discarded, and 10 mL water was added to the NDs pellets, and the mixture was sonicated to re-suspend and transferred to 10 Eppendorf tubes for ultrasonication (0-10 W, 60 s, 20 circles) while cooling with ice in between each interval of ultrasonication. Hielscher Sonicator (UP200St-G) equipped with S26d11x10 Vial-Tweeter-Sonotrode was applied for the powerful ultrasonication of NDs samples in Eppendorf tubes. NDs were also washed by centrifugation (10000 RPM, 30 min, 4 °C) and re-suspended in water after every five times of ultrasonication. The complete acid treatment combined with sonication (8 circles), and ultrasonication treatment (20 circles) was repeated three times. Finally, the NDs was re-suspend in 1 mL water to form a transparent colloidal dispersion, with removed non-suspended NDs by low-speed centrifugation (885 RPM, 10 min, 4 °C). Between and after sonication and acid treatment, the zeta-potential of NDs was measured to evaluate the efficiency of those treatments. As shown in Figure S8 and S9, negatively charged zeta potential with a single peak were obtained for NDs treated with multiple times of acid cleaning and sonication. In contrast, without sonication, the zeta-potential of NDs had multiple peaks, showing a non-uniformed surface charge for all facets of NDs and non-complete acid treatment. It is significant to perform sonication and powerful ultrasonication to achieve complete cleaning for all facets of NDs.

Preparation of polymer dcHSA-PEO

The polymer dcHSA-PEO for NDs coating was prepared according to reported protocols in three steps (Figure S4).[1]

1) Synthesis of cHSA

15 mg of HSA was completely dissolved in 1.5 mL of degassed ethylenediamine-HCl solution (2.5 M, pH = 4.75), followed by adding EDC (2000 equiv., 70 mg) and stirring for 75 min. The reaction was then terminated by adding acetate buffer (100 µL, 4 M, pH = 4.75). After the reaction, cHSA was washed twice with acetate buffer (100 mM, pH = 4.75) and three times with deionized distilled water by ultrafiltration (Vivaspin 20, 30 kDa MWCO) and then lyophilized to obtain cHSA as a white fluffy solid. The molecular weight of cHSA (72207.5663 Da) was identified by MALDI-Tof (Figure S11 a).

2) Synthesis of cHSA-PEO

cHSA (15 mg) was completely dissolved in degassed phosphate buffer (15 mL, 50 mM, pH = 8.0). MeO-PEG2000-NHS (35 equiv., 15 mg) was dissolved in 50 µL DMSO and added to the reaction mixture and stirring for two hours. After that, the reaction mixture was washed by ultrafiltration with ultrapure water eight, then lyophilized to obtain purified cHSA-PEO. MALDI-ToF result indicates a molecular weight increased to 99298.09 Da (Figure S11 b). An average increase in the molecular weight of 27090 Da was obtained indicating the attachment of about 13 PEG chains to the cHSA backbone.

3) Synthesis of dcHSA-PEO

cHSA-PEO (10 mg) was completely dissolved in 10 mL degassed urea-phosphate buffer (50 mM phosphate buffer, pH = 7.4, 5 M urea, and 2 mM EDTA) and stirred at the room temperature for 30 minutes, followed by adding TCEP (15 equiv., 0.43 mg) as solid and the solution and stirred for an additional 30 minutes under argon atmosphere. Then, N-(2-Aminoethyl) maleimide trifluoroacetate salt (100 equiv., 2.56 mg) was added and stirred overnight. After that, the reaction mixture was purified three times by ultrafiltration with urea-phosphate buffer (10 mM phosphate buffer pH 7.4, 5 M urea, and 2 mM EDTA) and five times with water. The

molecular weight of dcHSA-PEO (105882.0022 Da) was identified by MALDI-Tof analysis (Figure S12).

Preparation of polymer dcHSA-PEO coated ND-SiV (ND$_{SiV}$-polymer)

ND-SiV (1 mg) was dispersed in boric acid buffer (10 mL, 20 mM, pH = 8.4) and sonicated in a water bath for 10 min to form a homogenous suspension. dcHSA-PEO (4 mg) was dissolved in boric acid buffer (10 mL, 20 mM, pH = 8.4) and stirred at room temperature for 30 min. Then, NDs solution was added dropwise to the dcHSA-PEO solution (drop speed at 1 drop per 2s) and stirred at room temperature overnight (400 RPM). After that, the reaction mixture was concentrated by ultrafiltration to 1 mL; then the uncoated dcHSA-PEG was removed by centrifugation of ND$_{SiV}$-polymer (17000 RPM, 30 min, 4 °C) and re-suspended in water by sonication for more than three times. Then the ND$_{SiV}$-polymer were re-suspended in 1 mL water and stored at 4 °C.

Characterization of ND$_{SiV}$-polymer

1) Transmission electron microscope (TEM) measurement

ND$_{SiV}$-polymer solution (5 µL, 0.01 mg mL^{-1}) was dropped onto a freshly glow discharged 300 mesh size copper-grid covered with a continuous carbon film. The sample was dried at room temperature overnight. A Jeol 1400 transmissions electron microscope was used to obtain bright-field TEM images.

2) HR-TEM

HR-TEM imaging was conducted on an image-side corrected FEI Titan 80-300 operated at 80 kV.

3) Zeta potential measurement

ND-SiV, ND$_{SiV}$-polymer solution (0.5 mg mL^{-1}) were prepared in KCl solution (1 mM) and the zeta potential was recorded three times independently using Malvern Zetasizer ZEN3600 (Malvern Ltd, Malvern, UK).

4) Dynamic light scattering (DLS) measurement

DLS measurements were performed using a Malvern Zetasizer ZEN3600 (Malvern Ltd, Malvern, UK). ND-SiV solution was prepared at 0.5 mg mL^{-1} in water (ND-SiV in water), ND_{SiV}-polymer were prepared at 0.5 mg mL^{-1} in water and PBS solution (ND_{SiV}-polymer in water, ND_{SiV}-polymer in PBS). All three samples were measured at 25 °C with 173° angle. Autocorrelation functions were analyzed applying the cumulants method and COTIN routine to estimate the hydrodynamic diameter and polydispersity index (PDI). The hydrodynamic diameter was presented as a z-average value.

5) Fluorescence correlation spectroscopy (FCS) measurement

FCS measurements were performed on an Axiovert 200M – ConfoCor 2 system (Zeiss, Germany) using a C-Apochromat 40x, 1.2 W Corr. microscope objective (Zeiss, Germany). The excitation was done by a HeNe laser operating at 543 nm and the detected fluorescence light was passed through a long pass LP650 emission filter. Aqueous solutions of ND-SiV with a concentration of 0.5 mg mL^{-1} were studied at 23°C. For calibration, solutions of a reference dye with a known diffusion coefficient, i.e. Alexa 546 in water were also measured.

Live cell imaging by conventional confocal laser scanning microscopy

HeLa cells (human cervix carcinoma) were obtained from DSMZ (German Collection of Microorganisms and Cell Cultures, Braunschweig). HeLa cells were cultured in DMEM medium with high glucose supplemented and with 10% fetal bovine serum (FBS), 100 U mL^{-1} penicillin, 0.1 mg mL^{-1} streptomycin, and 0.1 mM non-essential amino acids at 37 °C in a humidified 5% CO_2 incubator. Thirty thousand Hela cells were plated in a µ-slide 8-well chambered coverslip (ibidi GmbH, Germany) in 300 µL medium. The cells were incubated overnight to allow adhesion. The next day, 5 µL of ND_{SiV}-polymer (1 mg mL^{-1}) were mixed with 250 µL cell culture medium to reach media concentrations of ND_{SiV}-polymer for 0.02 mg mL^{-1}. The cell culture medium and the ND_{SiV}-polymer mixture were sonicated for uniform

dispersion, and then the mixture was added to the Hela cells to replace the old cell culture medium. The cells were then further incubated for 24 hours in the incubator. Before imaging, the cells were washed three times with PBS buffer. Imaging was then performed using a Zeiss 710 confocal laser scanning microscopy under two channels. Channel 1, the excitation 561 nm with 80% laser power, the filter for fluorescence detection 700-758 nm. Channel 2, the excitation 561 nm with 5% laser power, the filter for reflection light detection 556-565 nm.

Dual-color cell imaging and fluorescence detection in presence of plasma membrane stain

Hela cells were seeded in an ibidi 8 well µ-slide (6×10^4 cells mL^{-1}, 300 µL in each well) and incubated overnight. After about 24h, old culture medium was removed and replaced with 100 µg mL^{-1} ND_{SiV}-polymer in culture medium with 300 µL each well. Next day the wells were washed 3 times with DPBS and added 300 µL fresh culture medium with CellMask Green diluted solution according to the manufacturer protocol. After 10 minutes 37°C incubation, the cells were washed two times with DPBS to remove the stain. Next, 300 µL fresh FluoroBrite DMEM was added before performing the microscopy at the room temperature using a customized scanning confocal microscope.

In the customized scanning confocal microscope (Figure S16), fluorescence was sent to an avalanche photo-diodes (APD, Excelitas) with high detection efficiency. The filter in front of the detector was long pass 575 nm to detect the CellMask green fluorescence. Later a band-pass filter with the transmission window of 698 - 766 nm was used to detect the signals from the ND_{SiV}-polymer. The confocal setup was operated under software Qudi (https://github.com/Ulm- IQO/qudi). The spectra were obtained by a spectrometer (Princeton Instruments, Acton SP 300i).

NDSiV-polymer for thermosensing in fixed cells

In an ibidi 8 well micro-slide, A549 cells were seeded with a concentration of 6×10^4 cells mL^{-1} and 300 µL in each well. After 24 hours of incubation, the old medium was replaced with

fresh cell culture medium containing 50 µg mL^{-1} ND_{SiV}-polymer (300 µL/well) and incubated overnight. Next day the cells were washed for three times with cold DPBS. Following 300 µL of DPBS containing 4% PFA (paraformaldehyde) was added for cell fixation. The cells were kept in room temperature for 20 minutes and washed for 3 times with DPBS. Afterwards 300 µL/well DPBS was added for cell imaging and spectra measurement. During the microscopy, the slide was kept in a stage-top incubator (OKOLAB H301-MINI) and maintained the desired temperature. The excitation was done by a 532 nm continuous-wave laser at 340 µW laser power measured in front of the objective. A spectrometer was used to record the fluorescence spectra under grating density 1800 g/mm, with acquisition time 5s. The same ND-SiV spot was repeatedly measured for 8 times at 25 °C and 37°C, separately. The ZPL data (730-750nm) was processed by Origin lab software (peak function-Lorentz fit) to obtain information of peak, FWHM, and intensity.

ND_{SiV}-polymer for thermosensing in living cells

In an ibidi 8 well micro-slide, A549 cells were seeded with a concentration of 6×10^4 cells mL^{-1} and 300 µL in each well. After 24 hours of incubation at 37°C, the old medium was replaced with fresh cell culture medium containing 50 µg mL^{-1} ND_{SiV}-polymer (300 µL/well) and incubated overnight. Next day the cells were washed for three times with DPBS. Afterwards 300 µL Gibco FluoroBrite DMEM was added to each well before cell imaging and spectra measurement. During the microscopy, the slide was kept in a stage-top incubator (OKOLAB H301-MINI) and maintained the desired temperature. The excitation was done by a 532 nm continuous-wave laser at 340 µW. A spectrometer was used to record the fluorescence spectra under grating density 1800 g/mm, with acquisition time 5s. The same ND-SiV spot was repeatedly measured for seven times at 25 °C and 37°C, separately.

The 3D position as well as the intensity of the ND-SiV spot were applied to identify the same ND-SiV spot at 25 °C and 37°C. The ZPL data (730-750 nm) was processed by Origin lab

software (peak function-Lorentz fit) to obtain information of peak, FWHM, and intensity (Figure S17). All the measured intracellular NDs-SiV with the same position similar intensity at 25 °C and 37°C showed thermo-resonance with red shift of the peak position of ZPL (Figure S17 a-d, g-j). For one of the ND-SiV spot in living cells (Figure S17 e-f), decreased intensity and blue shift of peak position of ZPL were observed. Due to the deceased intensity (about 35%), we can assume that the measured spots at 25°C and 37°C may not be the same ones therefore the measured spot was not considered as a suitable thermometry.

Cell imaging and nanodiamond tracking with customized scanning confocal microscope

The customized scanning confocal microscope was equipped with an oil-immersion (Nikon, NA=1.45, 100×). The excitation was done by a 532 nm continuous-wave laser. The fluorescence was divided by 50/50 beam splitter and sent to two avalanche photo-diodes (APD, Excelitas) with high detection efficiency. The filter in front of the first detector was long pass 575 nm. The second optical channel had a band-pass filter with the transmission window of 720-760 nm. The confocal setup was operated under software Qudi (https://github.com/Ulm-IQO/qudi). The spectra were obtained by a spectrometer (Princeton Instruments, Acton SP 300i).

The intracellular tracking was performed by refocusing every 40 s (Figure S18). Within the fluorescence image, a measurement loop was carried out repeatedly on single bright and photo-stable ND-SiV particles. Firstly, a fluorescence scan was performed in a small-range of 1.5 μm × 1.5 μm on the X-Y plane, to identify the position where maximum SiV fluorescence could be acquired on the plane. Based on this position, another scan of 2 μm on the Z-axis was performed to determine the final position where a maximum fluorescence intensity was found. This routine allows identifying the best focal point to collect maximum fluorescence counts in the well-aligned scanning confocal setup in the $1.5 \times 1.5 \times 2\ \mu m^3$. The optimized focal positions were saved as well as the maximum fluorescence intensities.

Supporting Figures

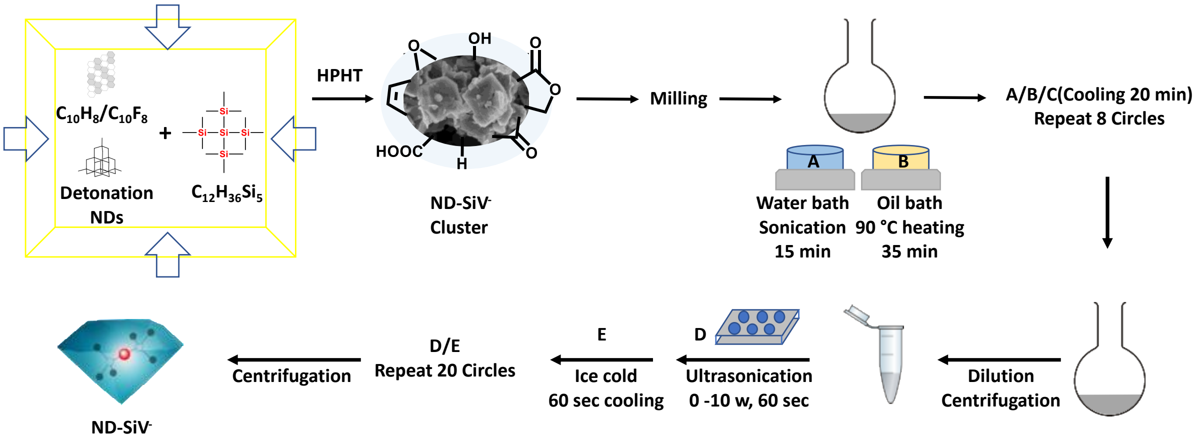

Scheme S1. Schematic presentation of ND-SiV HPHT synthesis and acid treatment. ND-SiV were produced from a homogeneous mixture of naphthalene ($C_{10}H_8$, Chemapol), octafluoronaphthalene ($C_{10}F_8$, Alfa Aesar), detonation NDs (3-4 nm, SkySpring), and tetrakis(trimethylsilyl)silane ($C_{12}H_{36}Si_5$, Stream Chemicals Co.).

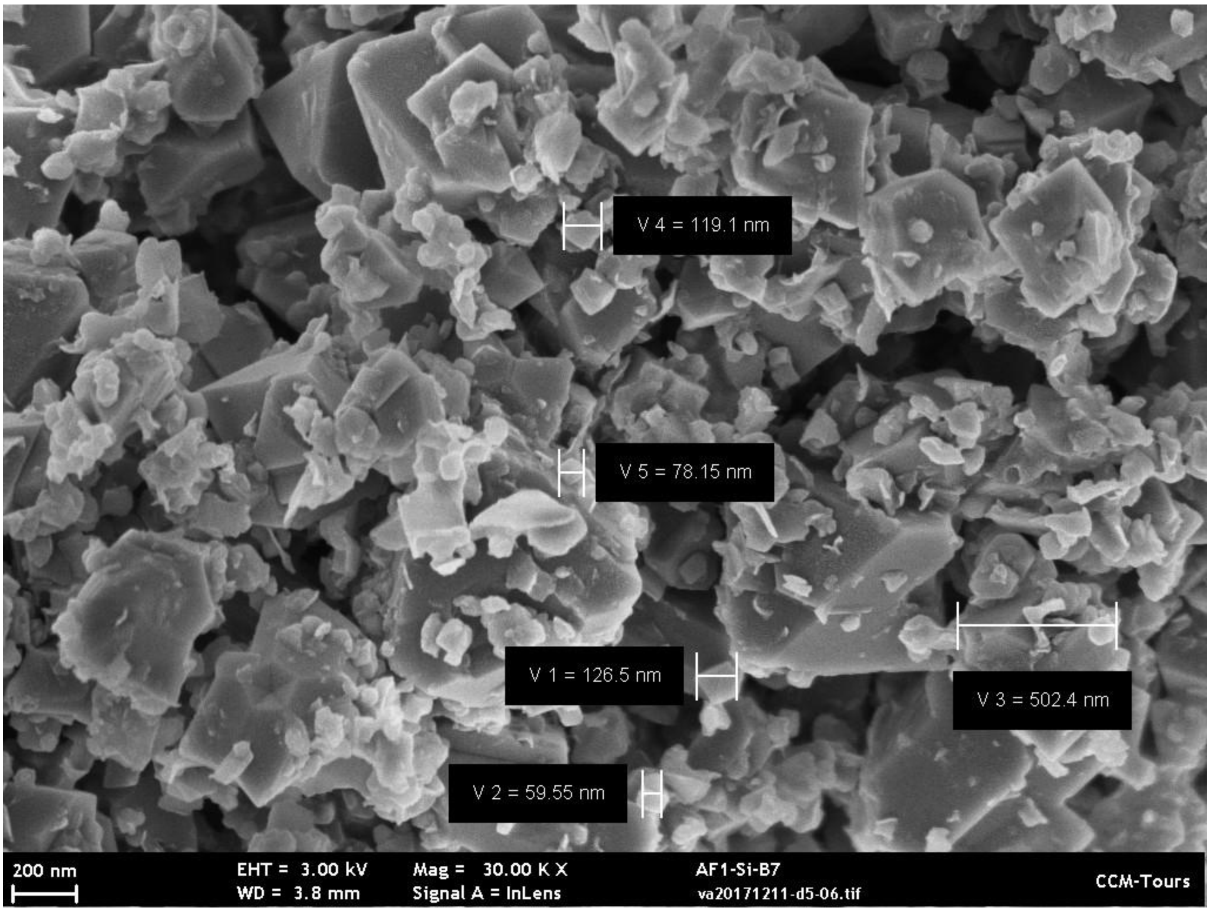

Figure S1. Scanning electron microscopy image of HPHT ND-SiV raw material.

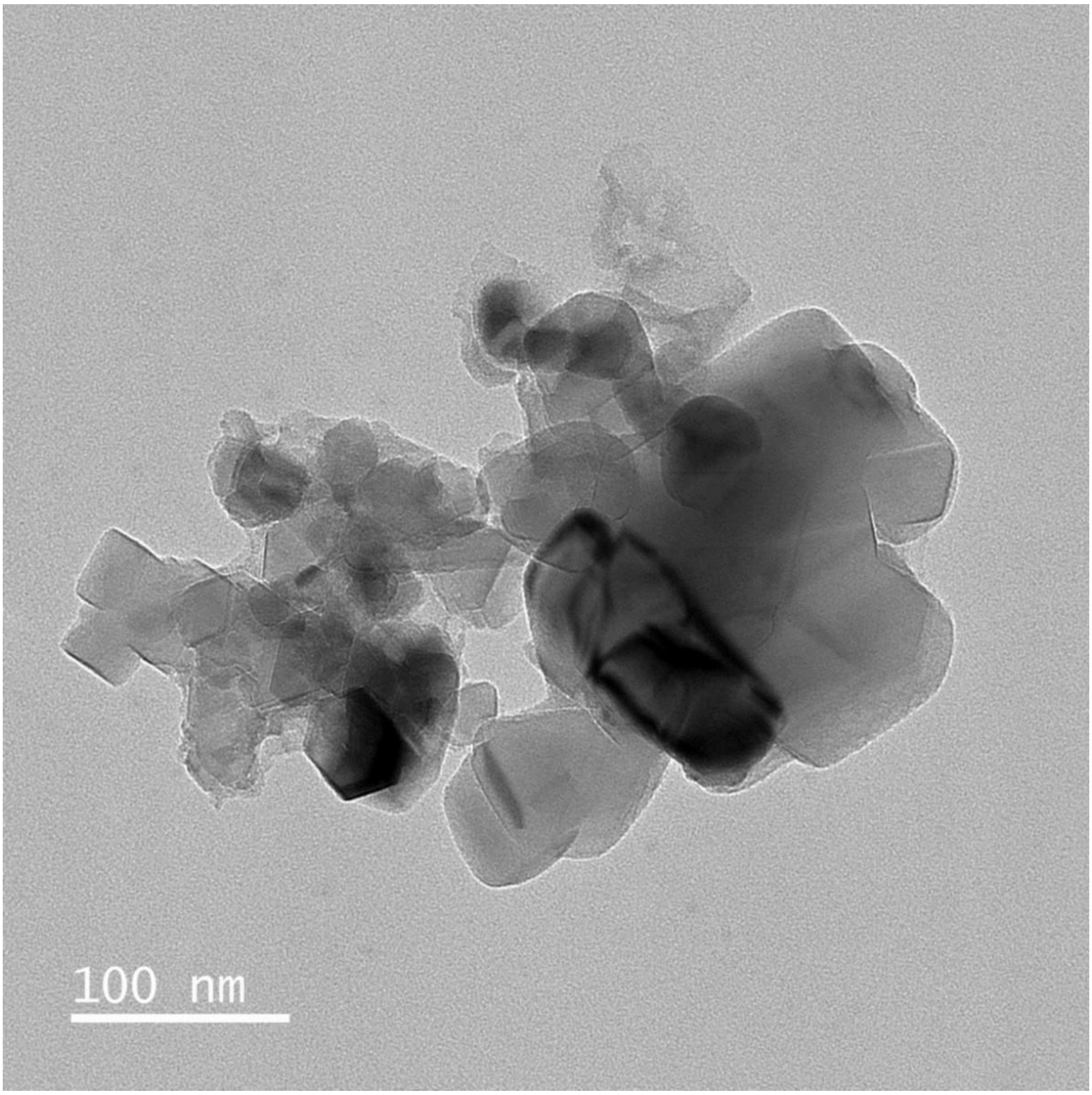

Figure S2. Transmission electron microscopy image of HPHT ND-SiV raw material.

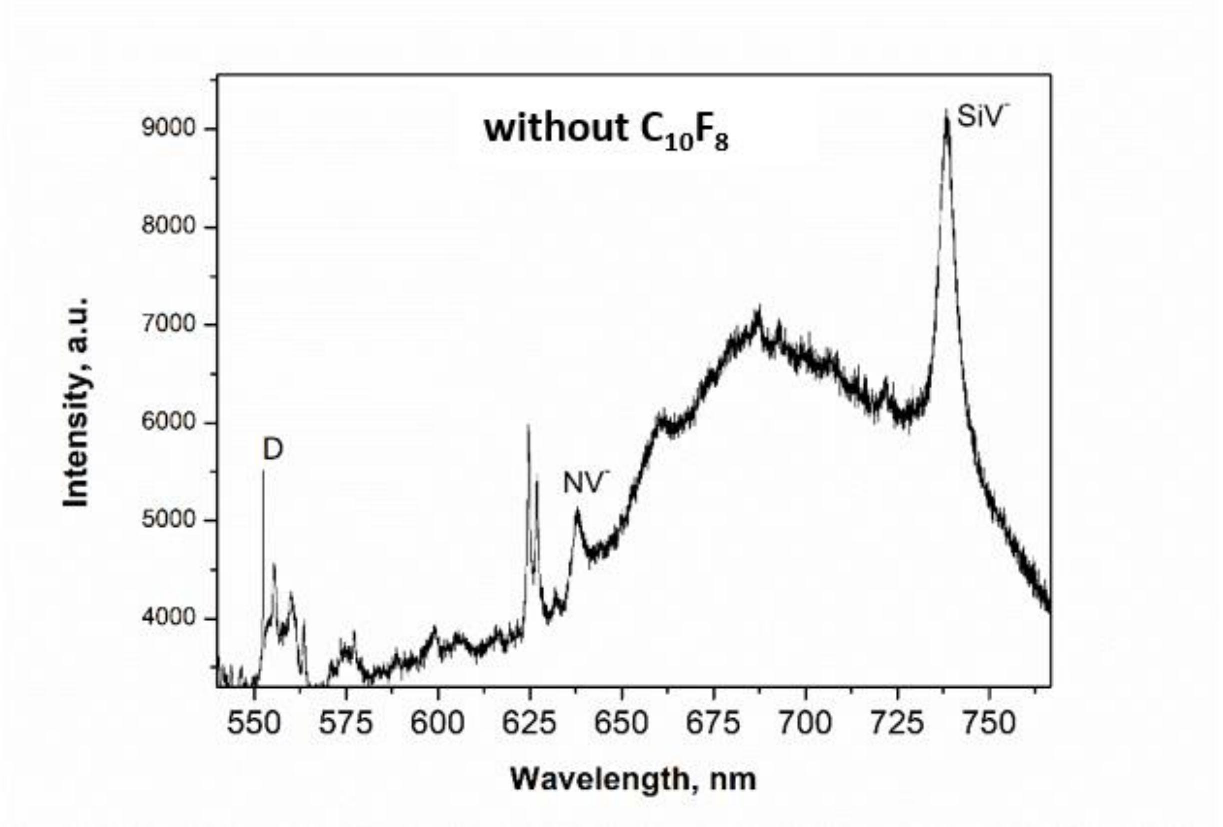

Figure S3. Photoluminescence spectrum of HPHT ND-SiV synthesized without $C_{10}F_8$.

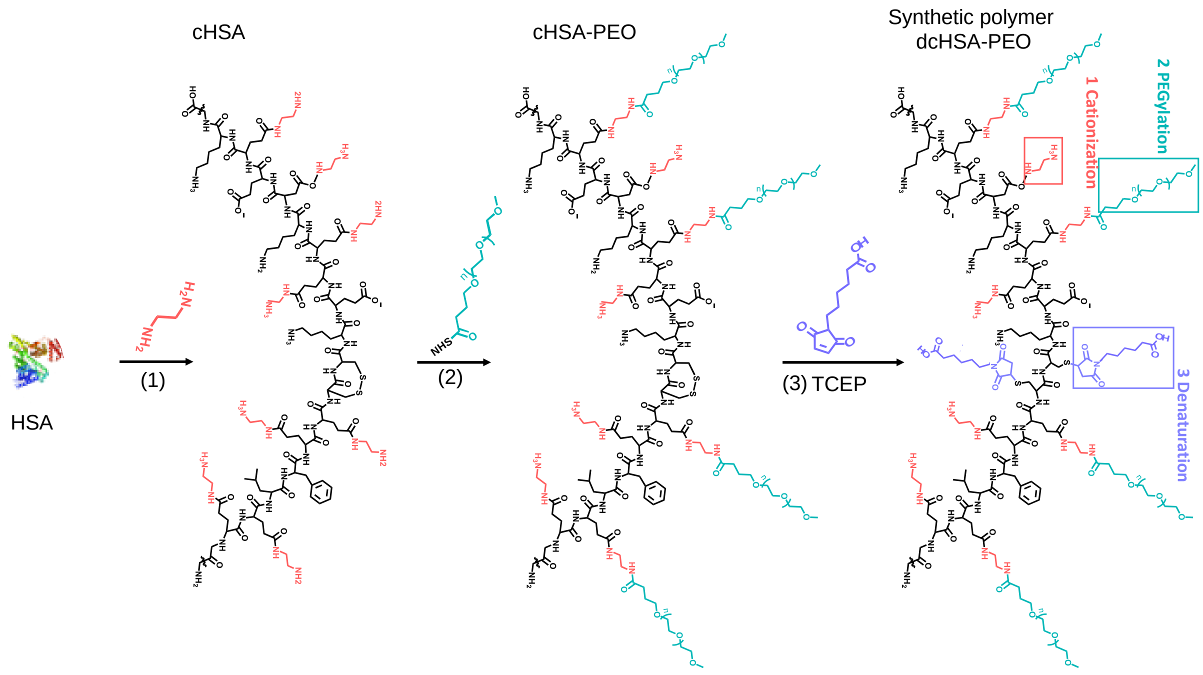

Figure S4. Three steps synthesis of dcHSA-PEO, step 1 cationization, step 2 PEGylation and step 3 denaturation.

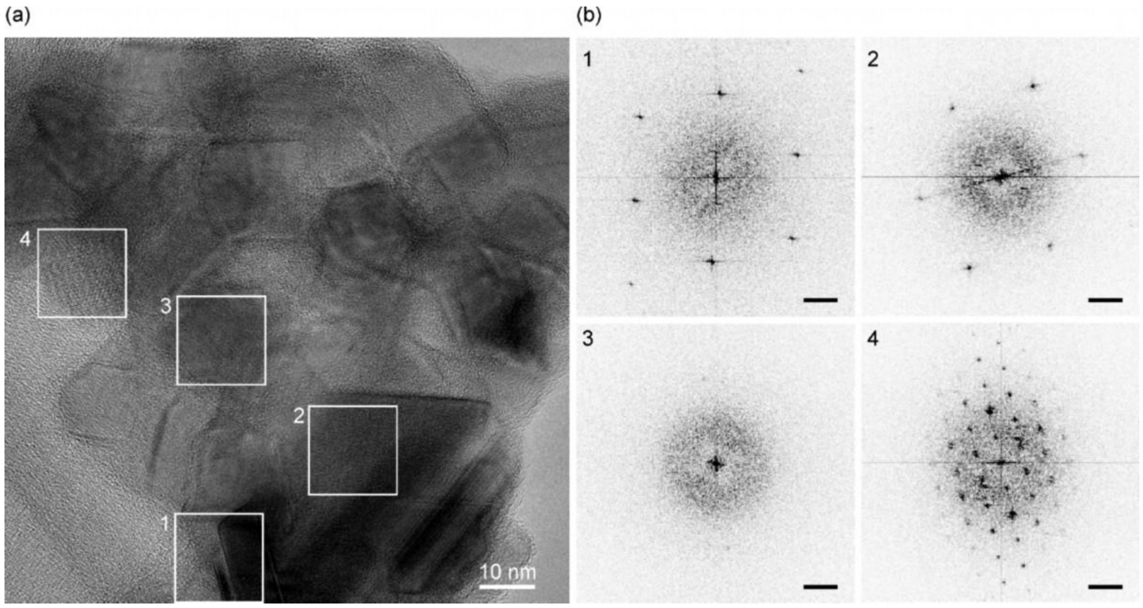

Figure S5. (a) AC-HRTEM image of a cluster of nanoparticles. (b) Fast-Fourier transform patterns of the corresponding regions marked by the squares in (a). Particles 1 and 2 are crystalline NDs, whereas particle 3 is amorphous. In region 4, a non-diamond crystalline particle is observed. Scale bar: 2 nm^{-1}.

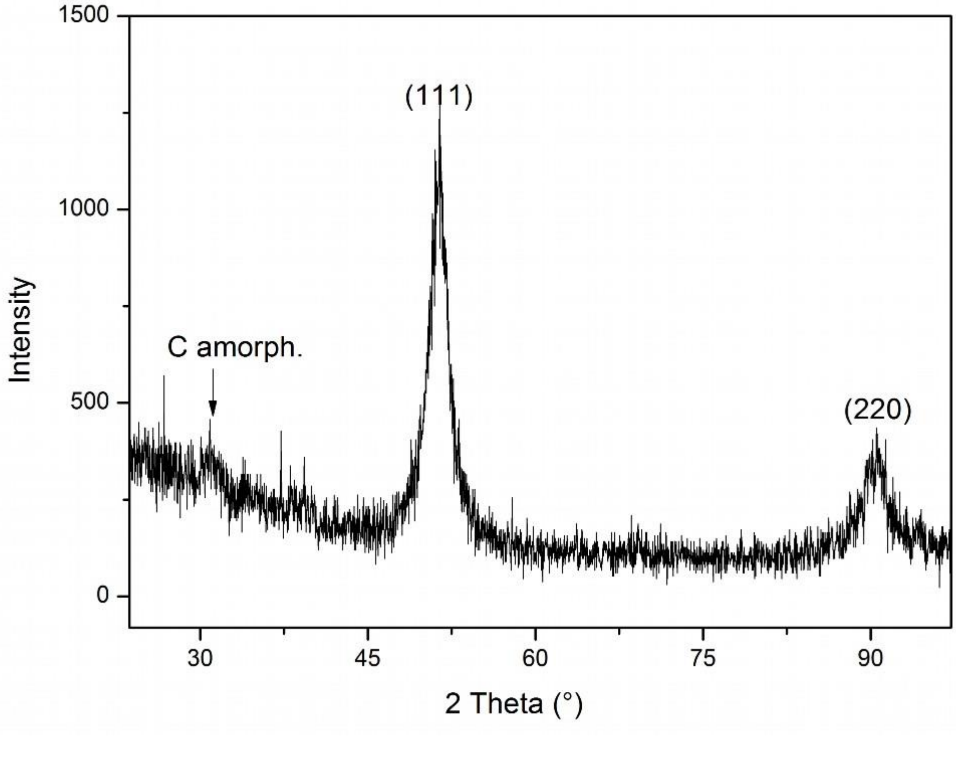

Figure S6. X-ray diffraction patterns (XRD) of ND-SiV raw material, which was recorded on an INEL CPS 120 diffractometer using Co Kα1 radiation source. The pattern was obtained in transmission mode.

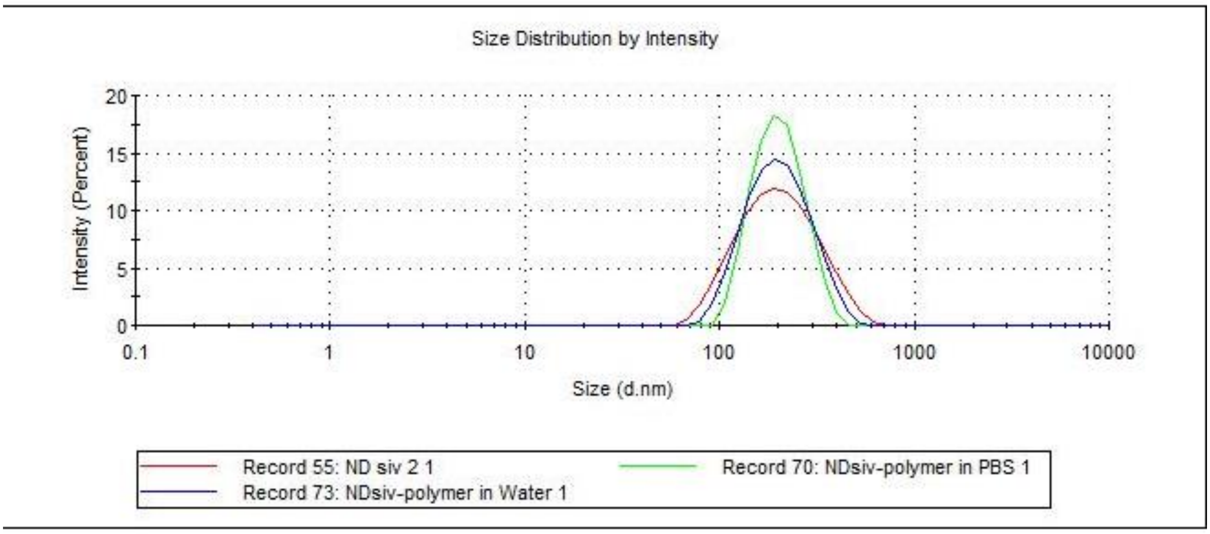

Figure S7. Dynamic light scattering size of ND-SiV and ND_{SiV}-polymer in water and PBS buffer.

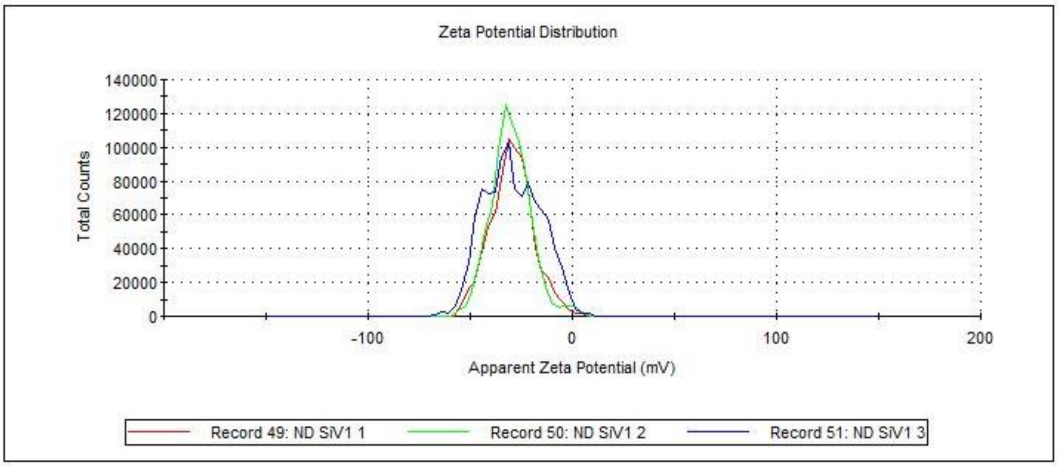

Figure S8. Zeta-potential of ND-SiV after acid treatment without multiple sonication steps.

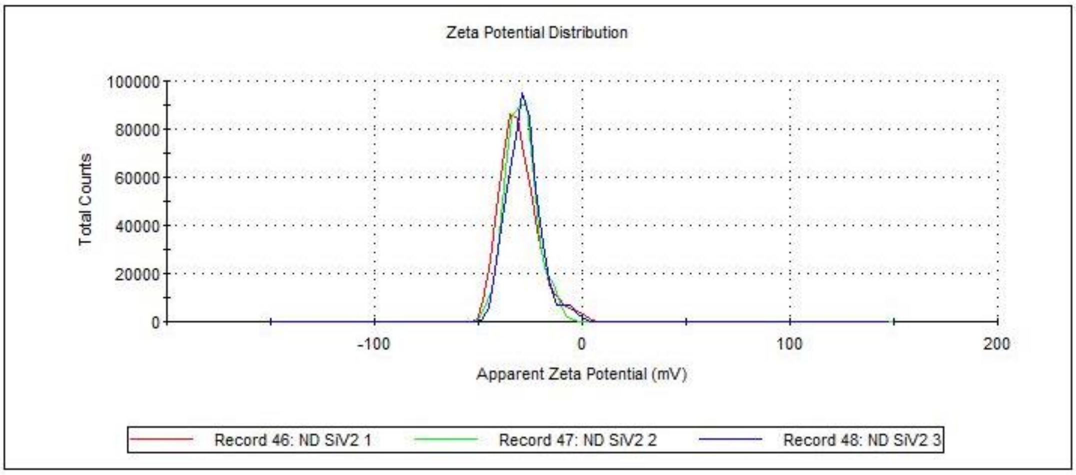

Figure S9. Zeta-potential of ND-SiV after acid treatment with multiple sonication steps.

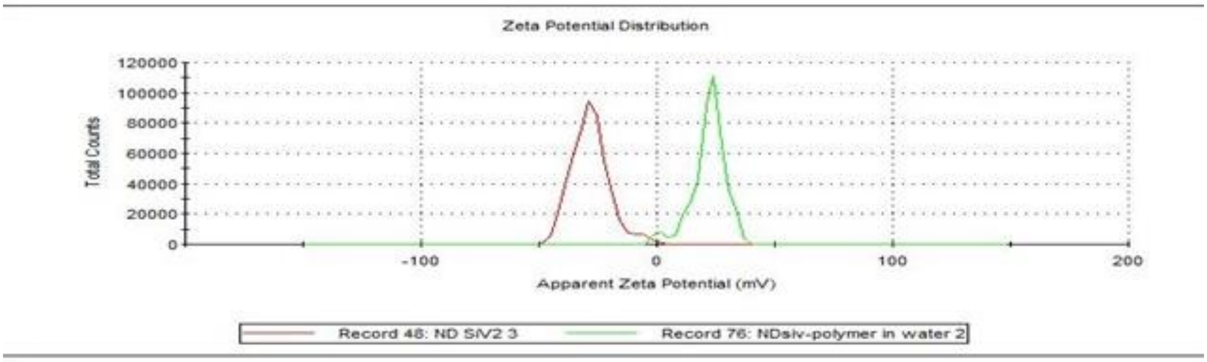

Figure S10. Zeta-potential of ND-SiV and ND_{SiV}-polymer.

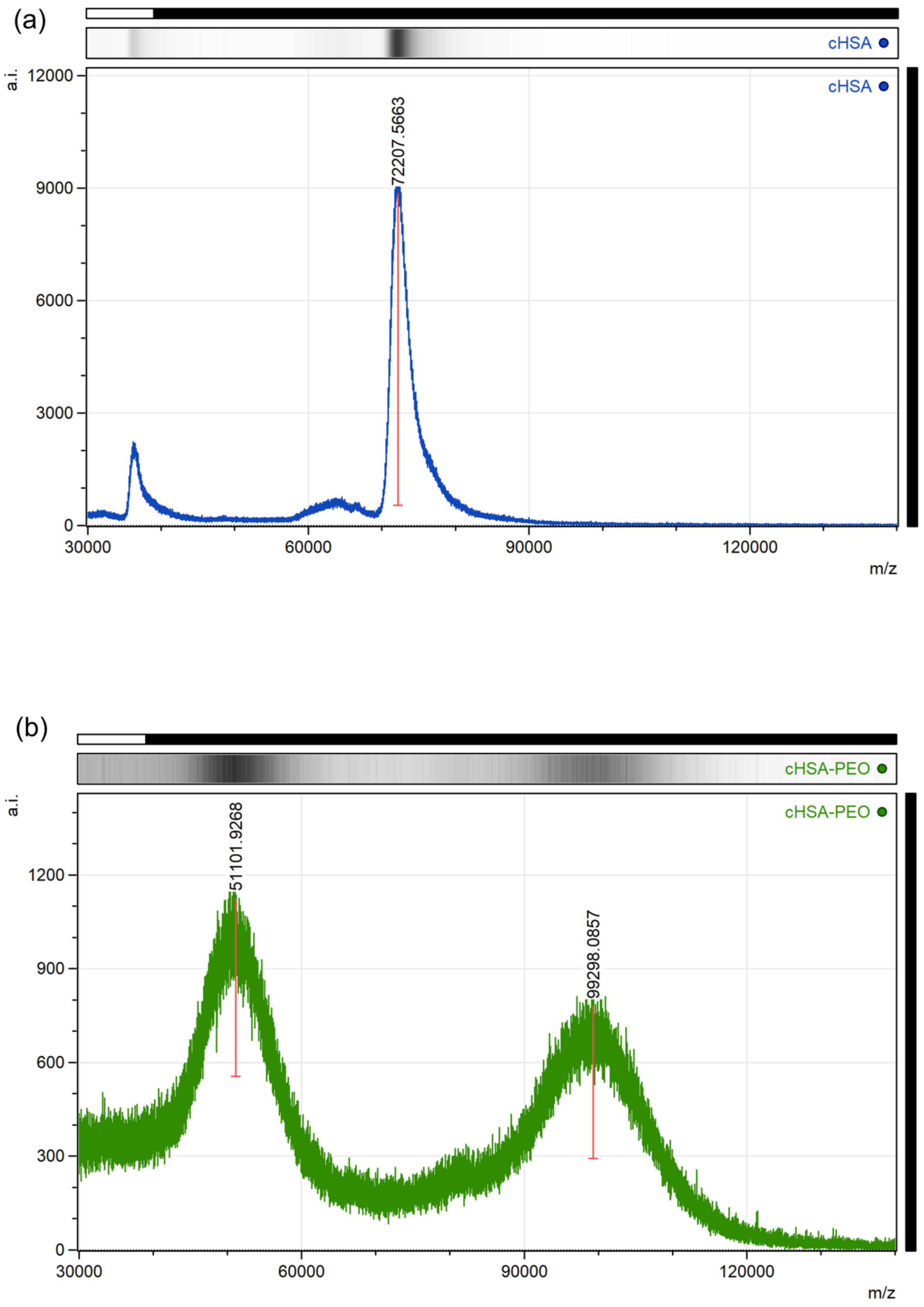

Figure S11. MALDI-Tof spectrum of (a) cHSA (72.2 KDa), (b) cHSA-PEO (99.3 KDa).

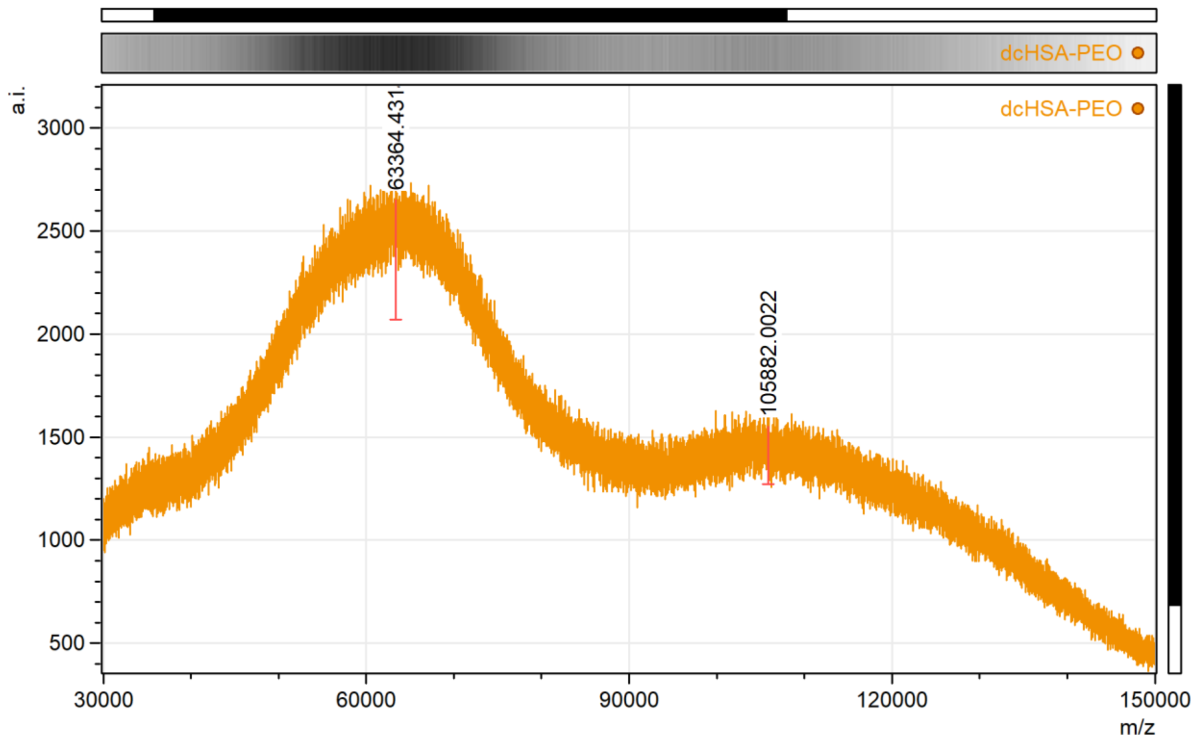

Figure S12. MALDI-Tof spectrum of dcHSA-PEO (105.8KD).

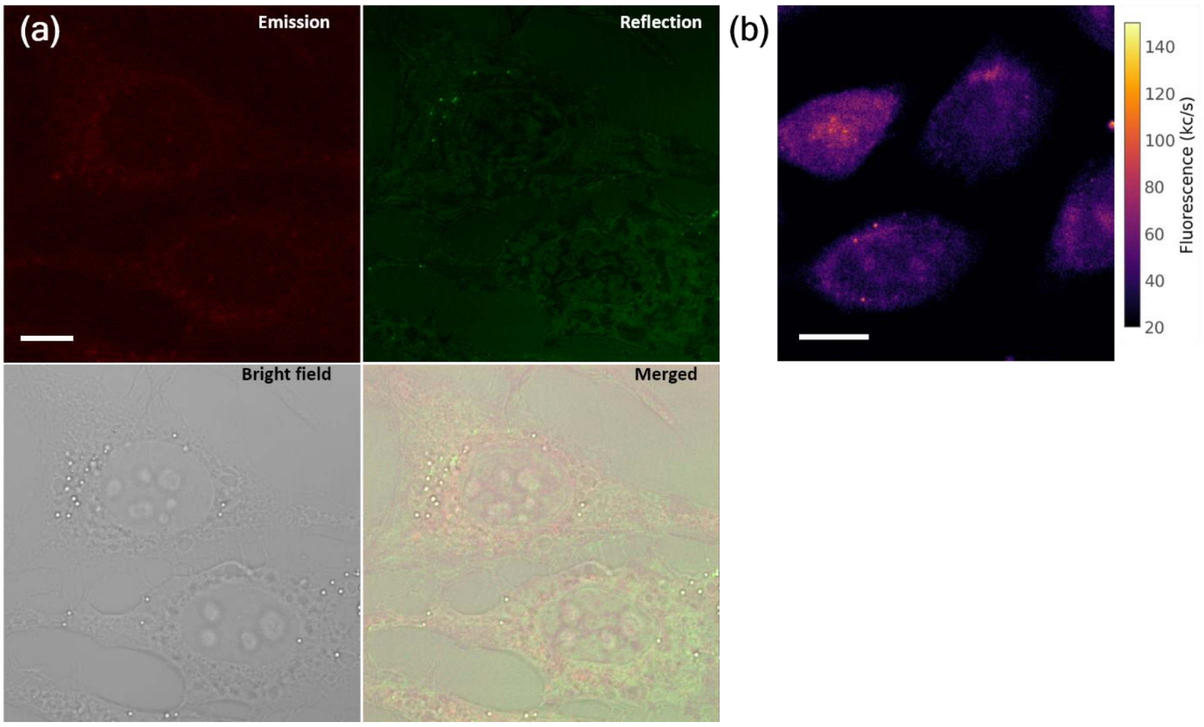

Figure S13. Blank control for (a) CLSM cell imaging and (b) customized scanning confocal microscope cell imaging (scale bar = 10 μm).

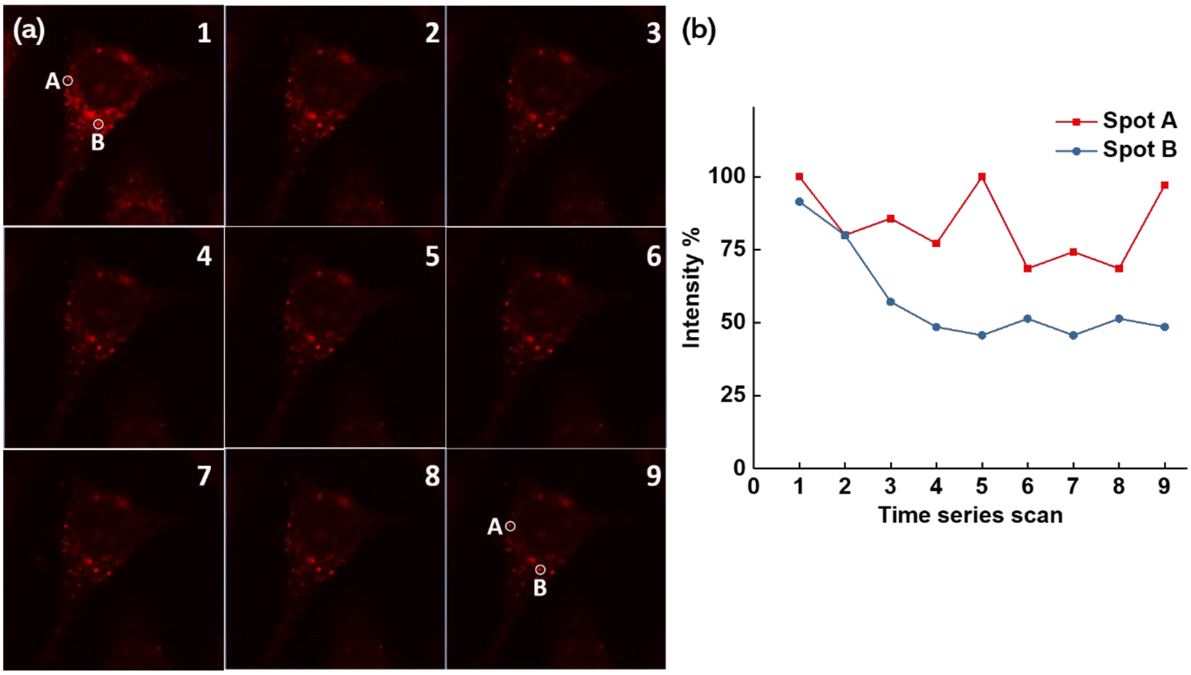

Figure S14. (a) Time series scan of cell images with ND_{SiV}-polymer taken up for photo bleaching test. Intensity of two fluorescent spots (marked as A and B) at each scan was recorded in (b).

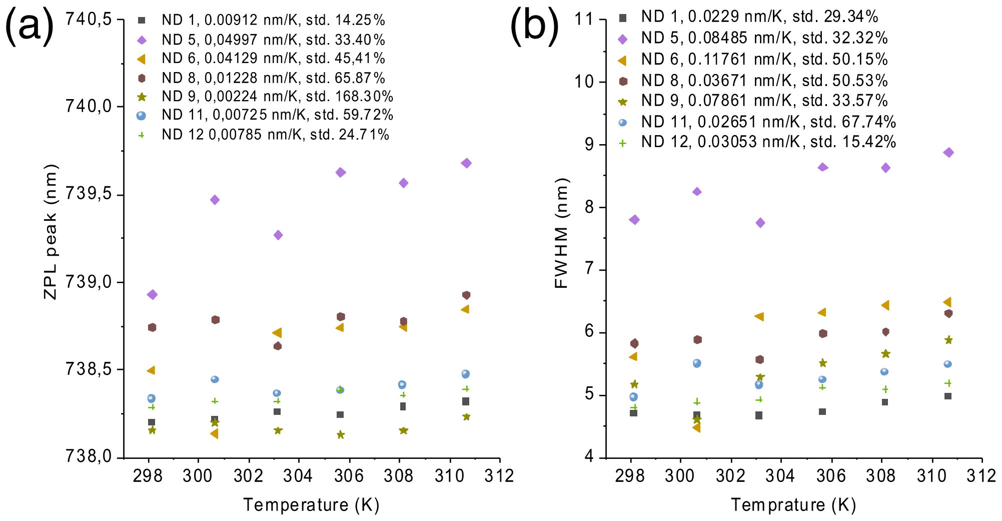

Figure S15. (a) ZPL peaks of 7 NDSiV nanoparticles with a non-linear shift in the temperature range of 25°C - 37.5°C with high deviation. (b) FWHM of ZPL spectra for the 7 NDSiV nanoparticles with a non-linear broadening in the temperature range of 25°C-37.5°C with high deviation.

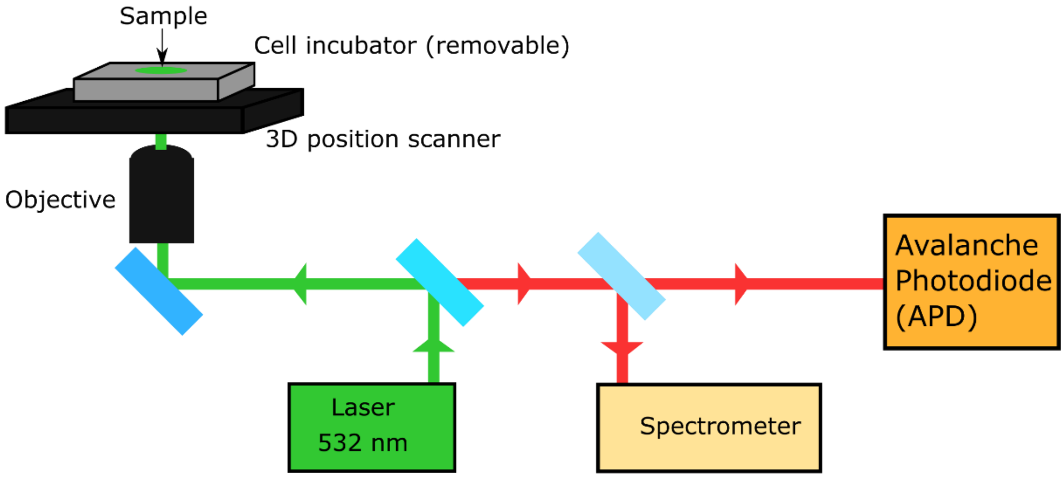

Figure S16. Scheme of the customized confocal microscope.

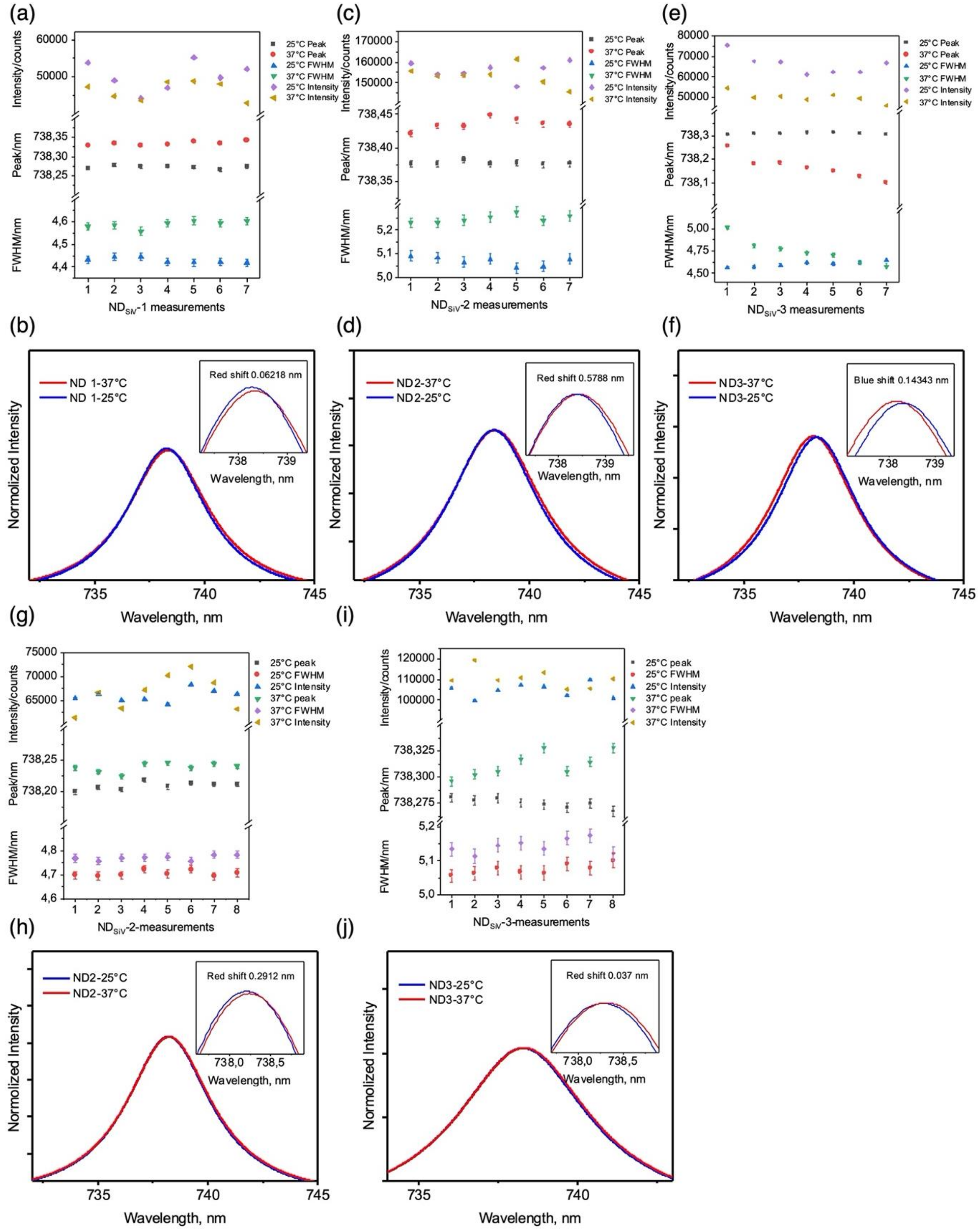

Figure S17. ND-SiV for thermosensing in living cells (a-f) and fixed cells (g-j). ZPL of the individual intracellular ND-SiV spot were repeatedly measured for 7 or 8 times at 25 °C and 37°C. For each measurement peak position of ZPL, FWHM, and intensity were analyzed.

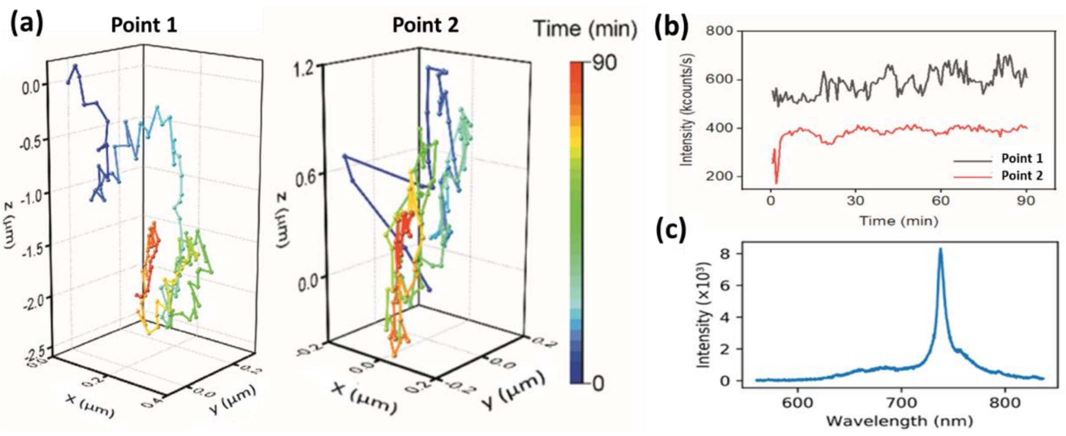

Figure S18. ND_{SiV}-polymer for cellular tracking. (a) The trajectory of two different NDs-SiV spots (point 1 and point 2) tracked in intracellular space. (b) Fluorescence intensity of the tracked NDs-SiV spots. (c) SiV spectra were observed from the NDs-SiV during the tracking measurements.

Reference:

(1) Wu, Y.; Ermakova, A.; Liu, W.; Pramanik, G.; Vu, T. M.; Kurz, A.; McGuinness, L.; Naydenov, B.; Hafner, S.; Reuter, R.; Wrachtrup, J.; Isoya, J.; Förtsch, C.; Barth, H.; Simmet, T.; Jelezko, F.; Weil, T. Programmable Biopolymers for Advancing Biomedical Applications of Fluorescent Nanodiamonds. *Adv. Funct. Mater.* **2015**, *25* (42), 6576–6585. https://doi.org/10.1002/adfm.201502704.
